# Broilers divergently selected for digestibility differ for their digestive microbial ecosystems

**DOI:** 10.1371/journal.pone.0232418

**Published:** 2020-05-18

**Authors:** Marion Borey, Jordi Estellé, Aziza Caidi, Nicolas Bruneau, Jean-Luc Coville, Christelle Hennequet-Antier, Sandrine Mignon-Grasteau, Fanny Calenge

**Affiliations:** 1 Université Paris-Saclay, INRAE, AgroParisTech, GABI, Jouy-en-Josas, France; 2 BOA, INRAE, Université de Tours, Nouzilly, France; Tokat Gaziosmanpasa University, TURKEY

## Abstract

Improving the digestive efficiency of broiler chickens (*Gallus gallus*) could reduce organic waste, increase the use of alternative feed not used for human consumption and reduce the impact of feed in production costs. By selecting chicken lines divergently for their digestive efficiency, we showed previously that digestive efficiency is under genetic control and that the two resulting divergent lines, D+ (high digestive efficiency or “digestibility +”) and D- (low digestive efficiency or “digestibility -”), also differ for the abundance of specific bacteria in their caeca. Here we perform a more extensive census of the bacteria present in the digestive microbiota of 60 chickens selected for their low apparent metabolizable energy corrected for nitrogen balance (AMEn-) or high (AMEn+) digestive efficiency in a [D+ x D-] F8 progeny of 200 individuals. We sequenced the 16S rRNA genes of the ileal, jejunal and caecal microbiotas, and compared the compositions and predicted functions of microbiotas from the different intestinal segments for 20 AMEn+ and 19 AMEn- birds. The intestinal segment of origin was the main factor structuring the samples. The caecal microbiota was the most impacted by the differences in digestive efficiency, with 41 bacterial species with abundances differing between highly and poorly efficient birds. Furthermore, we predicted that the caecal microbiota of efficient birds might be enriched in genes contributing to the degradation of short chain fatty acids (SCFA) from non-starch polysaccharides. These results confirm the impact of the genetic selection led on digestibility on the caecal microbiota taxonomic composition. They open the way toward the identification of specific, causal genes of the host controlling variations in the abundances of bacterial taxons.

## Introduction

Feed is one of the most important costs in broiler rearing, so that improving feed efficiency remains one of the main objectives of chicken breeders. The intense selection conducted during the last decades has led to a huge genetic progress when considering feed conversion and growth rate. This progress was obtained by feeding animals with optimal, easy-to-digest diets mainly composed of feedstuffs also used for human consumption. Selecting animals able to digest alternative sources of feed, which are more difficult to digest but not edible to human consumption could lead to a reduction in feed cost and would lead to a more durable breeding.

Broilers fed a diet difficult to digest, mainly comprised of a high-viscosity wheat variety (Rialto), display a vast variability in their digestive efficiency, measured as variations in nitrogen-corrected apparent metabolizable energy (AMEn) [1]. Mignon-Grasteau et al. (2004) [2] selected divergently two chicken lines for their digestive efficiency at 23 days of age when fed this diet, with 13% difference observed in AMEn between good digesters (D+) and poor digesters (D-), and 23.6%, 8.9%, and 5.9% differences observed respectively for lipid, starch and protein digestibility after one generation of selection. The difference in AMEn increased up to 25–30% after 8 generations of selection [3,4]. Several studies compared these divergent lines for parameters related to their digestive tract anatomy, histology and physiology. We hence know that the proximal part of the digestive tract of D+ animals, which show a better digestive efficiency, is more developed, with heavier gizzards and proventriculus. In contrast, the distal part of their digestive tract is less developed, with shorter intestinal segments (duodenum, ileum, jejunum). D+ animals also display a much longer retention time of feed in their proventriculus, gizzard and caeca, which increases the time for grinding, mixing with digestive enzymes and nutrient absorption [5]. As a result of these differences, efficient birds excrete a higher proportion of fine particles in their feces [5].

In addition to these physiological and anatomical differences between the D+ and D- lines, differences were observed in the abundances of specific bacteria species or groups in their caeca and ileum [6]. Abundances were quantified through targeted quantitative PCR on caecal samples from animals displaying contrasted FCR (feed conversion ratios) chosen among the F2 progeny of a D+ x D- cross. The species *Escherichia coli* and *Lactobacillus salivarius* were more abundant in animals with the highest FCR (lowest digestibility), while animals with the lowest FCR (highest digestibility) displayed higher *Clostridium leptum*, *Clostridium coccoides* and *Lactobacillus salivarius* to *E*. *coli* ratios, thus showing the potential impact of genetic selection led on digestibility on gut microbiota composition. Several other studies have shown that individuals differing for their feed efficiency harbor distinct microbiota compositions and identified specific OTUs associated with higher or lower feed efficiencies. Nevertheless, only a few studies suggested or showed the involvement of host genetics in these differences, by comparing lines divergently selected for growth or feed efficiency [6,7]. Besides, a few studies compared chicken lines divergently selected for adiposity [8,9], body weight, immune competence [10] and feather-pecking [11] and identified differences in the gut microbiota compositions of the divergent lines. Those results tend to show that the caecal microbiota of chicken is associated with the genetic control of many traits of interest.

In this study, we intended to gain more insights into the differences between digestive microbial ecosystems of D+ and D- animals, using the much more exhaustive approach based on 16S rRNA gene sequencing in an advanced intercross line of D+ and D- chickens. We selected animals based on their high or low coefficient of digestive use of dry matter (CUD_DM_) or AMEn, i.e. the criterion used for genetic selection and we studied three intestinal segments: jejunum, ileum and caeca. We also predicted from the abundance tables obtained the putative differences in functions carried out by the digestive microbiotas of animals with high or low levels of digestive efficiency. Our main objectives were to (i) assess potential differences in the intestinal microbiota composition between groups of highly efficient vs poorly efficient birds, in three distinct intestinal segments and (ii) to infer putative differences in the functional pathways realized by the intestinal microbiota between these animals.

## Materials and methods

### Animal rearing

The divergent lines D+ (higher digestibility) and D- (lower digestibility) were genetically selected according to their apparent metabolizable energy corrected for zero nitrogen (AMEn) at 25 and 26 days, from a commercial population of a medium growing broiler line, as previously described [2]. For the present experiment we reared a F8 AIL (advanced intercross lines) progeny of 200 animals deriving from initial crossings between D+ and D- animals [12] followed by crossings between animal of distinct families at each generation until the 8^th^ generation considered here, according to the recommended breeding scheme for AIL [13]. The AIL F8 progeny were reared at the PEAT Poultry Experimental Facility (INRAE, Nouzilly; https://doi.org/10.15454/1.5572326250887292E12), first on floor covered with fresh wood chips during the first week, in order to favor a normal installation of the digestive microbiota, and then in individual cages to be able to collect their feces. Animals were equally distributed into 3 independent pens in the same building. Animals were fed *ad libitum* with the same challenging diet used during the original selection experiment, i.e. a diet adapted to nutritional requirements (2943 kcal/ kg of dry matter, 21% of proteins; 6% lipids) but of low digestibility due to its high content (55%) in a wheat variety with high viscosity (Rialto) (S1 Table). This diet emphasizes differences in individual faecal digestibility values [2]. All animal care and experimental procedures needed for this study were conducted in accordance with the French Animal Welfare Act and were approved by the Ethics Committee for Animal Experimentation of Val de Loire (Authorisation No. 01047.02). This ethics committee is registered by the National Committee under the number C2EA-19.

### Sample collection

We analyzed the digestive microbiota from a subset of 60 birds with extreme digestive efficiency values. Due to the long time required for AMEn determination, it was necessary to find a proxy of AMEn at 3 weeks to select these birds. Previous analyses showed that coefficient of digestive use of dry matter (CDU_DM_) and AMEn were strongly correlated at 3 weeks. We thus made a first balance trial between 13 and 15 days using a method based on total excreta collection, as described previously [14]. We calculated CDU_DM_ at 2 weeks as a proxy of AMEn at 3 weeks as follows:

$${CDU}_{DM}=\frac{{FI}_{DM}-{FW}_{DM}}{{FI}_{DM}}$$

where FI_DM_ is the dry matter feed intake between 13 and 15 d and FW_DM_ the dry matter feces weight between 13 and 15 d. Among the initial population (N = 213), we thus selected two subsets of 30 birds (males and females) displaying respectively the lowest and highest values of CDU_DM_ at 2 weeks. These two groups will be called CDU_DM_+ and CDU_DM_-. We performed a second balance trial between 25 and 26 d to obtain fecal AMEn and coefficients of digestive use of dry matter (CDU_DM_). For this second phenotyping, titanium dioxide was used in the diet (0.5%). Digestive efficiency traits were calculated as follows:

$${CDU}_{DM}(\%)=100-\frac{{TD}_{d}}{{TD}_{f}}\times 100$$

$$AMEn={RE}_{d}-\left(\frac{{RE}_{f}\times {TD}_{d}}{{TD}_{f}}\right)-82.2\times \left(N_{d}-(\frac{N_{f}\times {TD}_{d}}{{TD}_{f}})\right)$$

where TD_d(f)_ is the titanium dioxide content in the diet (feces), S_d(f)_ the starch content in the diet (feces), FA_d(f)_ the fatty acids content in the diet (feces), N_d(f)_ the nitrogen content in the diet (feces), UA_f_ the uric acid content in the feces, RE_d(f)_ the raw energy content in the diet (feces).

At 27 days of age, animals were euthanized by an intra-veinous injection of pentobarbital (1,5 mL/ kg). We gently removed intestinal contents from the intestines so as not to collect the mucosa. The content of distal ileum defined as the two-thirds distal intestinal segment between the Meckel’s diverticulum and the ileo-caecal junction without the two last centimeters near the junction were sampled and homogenized. We also sampled the content of the jejunum, and the content of both caeca, which we homogenized. All intestinal contents were snap-frozen in liquid nitrogen and stored at −80°C until DNA extraction.

### DNA extraction, amplification and sequencing

We carried out subsequent analyses on the two subsets of animals with extreme CUD_DM_. We extracted the DNAs from 200 mg of their jejunal, ileal and caecal contents using a standardized protocol slightly adapted from Godon et al. [15]. Briefly, 200 mg of each sample were suspended in a buffer containing Guanidine Thiocyanate (SIGMA ALDRICH) and N-Lauroyl Sarcosine (SIGMA ALDRICH) and incubated for 1 h at 70°C. Sterile silica beads (0.1 mm) were used for bacterial cell lysis with a vibro mill at 25 stirring.sec^-1^ for 10 min (Retsch MM200) bead beater (MP biomedicals). We then added 20 mg of Polyvinylpyrrolidone (SIGMA ALDRICH) and the suspension was vortexed and centrifuged (14000 g, 4°C, 3 min). The supernatant was recovered, the pellet was washed with 500 μL TENP buffer, centrifuged again and washed two more times. The pooled supernatants were centrifuged (14000 g, 4°C, 10 min). Nucleic acids were precipitated in isopropanol. After 10 min incubation at room temperature, the mixture was centrifuged (14000 g, 4°C, 10 min) and the supernatant was removed. The pellet was resuspended in 270 μL of phosphate buffer 0.1 M (pH 8) (SIGMA ALDRICH) and 30 μL of potassium acetate 5 M (Euromedex) and left at 4°C for 2 h. We added 2 μL of RNAse (10 mg/mL) and incubated it at 37°C for 30 min. DNA was precipitated using 50 μL of 3 M sodium acetate and 1 mL of ice cold 100% ethanol. After incubation at −20°C for 1 h the DNA pellet was washed three times with 70% ethanol, dried and stored at 4°C in 300 μL TE buffer.

DNA quantities were evaluated using a Qubit analyser, and DNA qualities using a Nanodrop analyser by measuring ratios of absorbances at 240, 260 and 280 nm. We diluted DNA samples to reach a concentration of 15 ng/μL before PCR amplification. A 464 bp fragment targeting the hypervariable V3-V4 region from bacterial 16S rRNA gene was first amplified with the following primers: PCR1F_343 (5’-CTTTCCCTACACGACGCTCTTCCGATCTACGGRAGGCAGCAG-3’), and PCR1R_784 (5’-GGAGTTCAGACGTGTGCTCTTCCGATCTTACCAGGGTATCTAATCCT-3’), in accordance with primer adapter previously reported [16]. We conducted the first PCR reaction with 1 μL of genomic DNA and 0.5 μL of each primer (10 μM), 0.5 μL of dNTP mix (10 mM), 2.5 μL of 10X MTPtaq buffer mix (10 mM), 0.25 μL of MTP Taq DNA Polymerase (SIGMA-ALDRICH) and H2O qsp 25 μL. PCR conditions were: initial denaturation at 94°C for 1 min, followed by 30 cycles of 94°C for 1 min, annealing at 65°C for 1 min, extension at 72°C for 1 min and final elongation step at 72°C for 10 min. We checked by agarose gel electrophoreses that each PCR generated a unique product at the expected fragment length. DNA Purifications, second PCR with the same primers extended by Illumina adapters, and sequencing using a 2x300 bp MiSeq Illumina sequencer were performed within the GetPlaGe platform (INRAE, Toulouse).

### Bioinformatic analyses

We used a Qiime v1.9 pipeline to assess microbial composition and abundances from the raw 16S rRNA gene sequences [17,18] by using the Subsampled open-reference OTU clustering approach according to the authors recommendations. In brief, we first controlled the raw sequences for their quality and aligned them to the GreenGene13_8 database to annotate them based on a 97% identity threshold. Then we performed a *de novo* assembling of those of the sequences that failed the first annotation; we used Uclust and aligned the obtained OTUs on the GreenGene13_8 database for a second round of OTU identification. We merged the two sets of OTUs, eliminated chimera OTUs and used the trimmed list of OTUs to build an abundance Table and a phylogenetic tree.

### Statistical analyses

We performed statistical analyses of microbial composition of our samples using R 3.5.2 [19].

#### Exploratory analyses

We first cleaned the data by filtering out singletons and rare OTUs (<0,005% reads) [20]. Rarefaction was conducted once on the full data set. From the distribution of OTU counts, we chose a conservative threshold of a minimum of 2500 reads to keep OTUs in our Table. Using the Vegan R package, we calculated the alpha- (Shannon index) and beta-diversity indices, and the richness of the whole set of data after rarefaction. Non-Metric Multidimensional scaling (NMDS) plots based on Bray-Curtis distance were used as a first exploratory analysis, to identify the factors significantly influencing the distances between samples (namely sex, digestibility level and intestinal segment). Since the segment of origin was by far the most important factor stratifying microbiota samples, we decided to conduct subsequent analyses separately for each intestinal segment. For each segment independently (ileum, jejunum, caeca), we assessed the relative composition in OTUs at different taxonomic levels. We then calculated alpha- and beta-diversity indices, and richness.

#### Assessing differentially abundant OTUs between digestibility groups

Then we used the metagenomeSeq package. Raw OTU counts were normalized through a cumulative-sum scaling (CSS) method to reduce biases due to uneven sequencing depth [21]. This allowed us to compare OTU abundances between the two groups of extreme animals AMEn+ and AMEn-, using a zero-inflated Gaussian mixture model with digestibility level (D+ vs D-) and sex as main effects, in order to identify OTUs differentially abundant (adjusted P-value<0.05) between AMEn+ and AMEn- groups in each intestinal segment independently (ileum, jejunum, caeca). We performed analyses independently at different taxonomic levels: species, genus, family. At each taxonomic rank, we agglomerated OTUs from the same family or genus using the Phyloseq R package.

#### Prediction of putative functions carried by OTUs and identification of differentially abundant functions between AMEn+ and AMEn- samples

We predicted putative microbial gene functions from caecal, jejunum and ileal microbiota using OTU abundance tables with the PICRUSt 1.1.0 package [22]. We calculated Nearest Sequenced Taxon Index (NSTI) to assess the similarity between the studied microbial communities and the PICRUSt metagenome reference database in each segment. Using the Kyoto Encyclopedia of Genes and Genomes (KEGG) database as genome function reference, we thus obtained a prediction of KEGG Orthology (KO) abundances in caecum and ileum. KO abundances for each sample were then explored for differential abundance by using the DESeq2 1.22.2 R package, with the animal sex as co-variable, and the digestibility group (AMEn+ vs AMEn-) as main variable.

We then performed a Wald test to identify sets of KO functions significantly more abundant in either AMEn+ or AMEn-, in either caeca or ileum, with a p-value adjusted to 0.05 (Benjamini-Hochberg). We then used the clusterProfiler R 3.10.1 package to obtain the pathways, KEGG identifiers, and abbreviations corresponding to these two sets of KEGG functions.

In parallel, the PICRUSt software enabled us to obtain, for each specific KEGG function, the sample contribution of each OTU through the metagenome_contributions.py pipeline. For each identified differentially abundant function between AMEn+ and AMEn- samples, we could thus predict the contribution of each putative OTU genome to obtain a Table with the count contributed by OTU for each sample. OTUs that appeared in more than 50% of the samples per tissue and digestibility group, and with at least 100 counts in one sample of the ‘CountContributedByOTU’ column were selected to obtain a graphic illustration of the average count of each identified KEGG function and of the main OTU contributing to these predicted functions. We only observed three KEGG functions with differential abundance between each digestibility group for the jejunal samples. Furthermore, none of these functions was related to metabolism. Concerning ileum samples, every differentially abundant function was more abundant in the AMEn- group. Consequently, we discarded jejunum AMEn+, jejunum AMEN- and ileum AMEn+ groups from further analyses on functional predictions. To complete these illustrations, corresponding tables for caecal AMEn+, caecal AMEn-, and ileum AMEn- group with each KEGG identifier name, fold-change and adjusted P-value from deseq2 statistical analysis are available in supplementary data (S2 Table).

## Results

### Digestibility phenotyping and establishment of contrasted groups

The mean AMEn value at 25 and 26 days of the 213 F8 chicks was 2752.5 ± 315.7 kcal.kg^-1^ DM. Although previous work showed strong positive correlations between AMEn and CUD_DM_ at 23 days, we could not observe such a correlation between CUD_DM_ at 14 days and AMEn at 23 days. We thus had to select, within the initial subset of 60 animals, another subset of 39 animals differing for their AMEn at 25 and 26 d and not on their CDU_DM_ at 14 d. From this subset, there were 22 males and 17 females equally distributed in the 3 pens (S3 Table). Neither sex nor pen had any significant effect on digestibility (chi-square test, resp. p-value of 0.897 and 0.054). We were able to select one set of 20 AMEn+ animals with a mean AMEn of 3201.2 ± 47.2 kcal.kg^-1^ DM and one set of 19 AMEN- animals with a mean AMEn of 2573.4 ± 366.7 kcal.kg^-1^ DM. The D+ and D- lines displayed comparable mean values after the first round of genetic selection, i.e. 3308 ± 251 kcal.kg^-1^ DM and 2921 ± 249 kcal.kg^-1^ DM, respectively [2]. Finally, the set of 19 AMEn- animals chosen displayed AMEn values representative of the variability usually displayed by the D- line, which shows a much greater variability in AMEn values than the D+ line.

### Exploratory microbiota analysis over all intestinal segments

**Fig 1:**
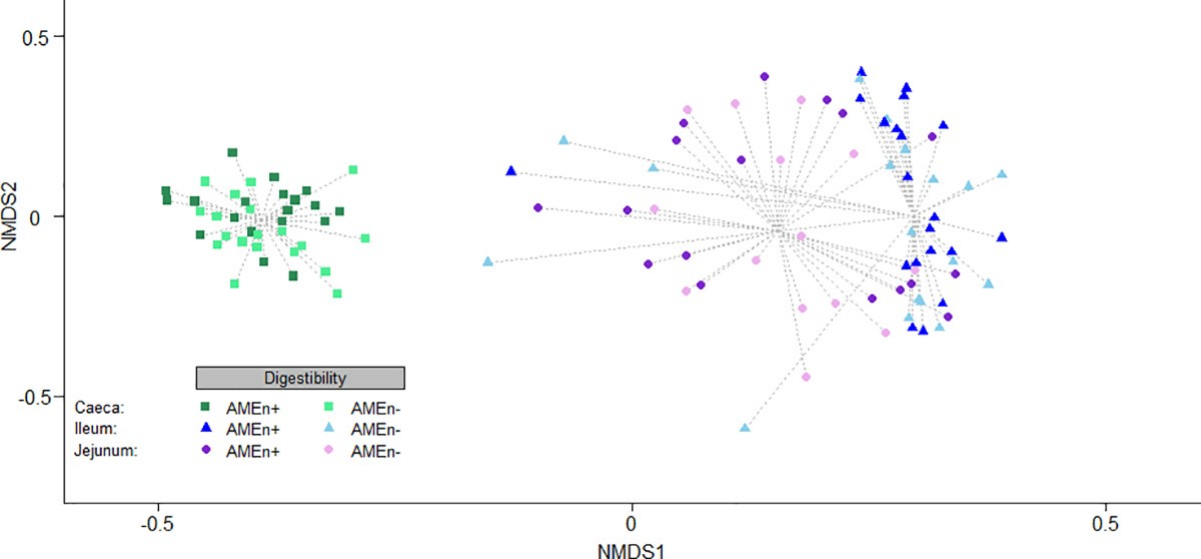
NMDS representation of Bray-Curtis distances between samples calculated from OTU counts, according to their intestinal segment of origin; Ca: caeca; Il: ileum; Je: jejunum.

Raw sequences are available through the accession PRJNA611508 on the NCBI SRA (Sequence Read Archive) (https://www.ncbi.nlm.nih.gov/sra/PRJNA611508). We obtained an average of 59,754 raw sequences per sample, while the total count for each OTU over all the samples ranged between 1,344 and 193,349. Due to the rarefaction step to 2500 reads, 10 samples with less than 2500 reads had to be discarded, over the 39 * 3 samples considered. Thus from the 39 animals initially selected for the study, we performed the microbiota analysis over 38 samples in the ceaca and ileum, and 31 in the jejunum. We identified 512 OTUs, reduced to 359 after filtering. We observed 489 OTUs in caeca, 478 in jejunum and 425 OTUs in ileum after filtering. The Bray-Curtis NMDS analyses identified the intestinal segment of origin as the main driver of OTU stratification among samples (Adonis p-value = 0.0001), which is illustrated by the distinct clusters obtained for samples from jejunal, ileal and caecal segments (Fig 1). Neither sex nor pen displayed any significant effect on microbiota alpha and beta-diversity in any of the three intestinal segments (S4 Table, S1 and S2 Figs). When considering intestinal segments separately, digestibility level (AMEn^+^ vs AMEn^-^) had a significant effect only in caeca (Adonis p-value_caeca_ = 0.003, p-value_Ileum_ = 0.34, p-value_jejunum_ = 0.82) (Fig 1).

### Microbiota differences between three intestinal segments

#### Alpha-diversity, beta-diversity and richness

**Fig 2:**
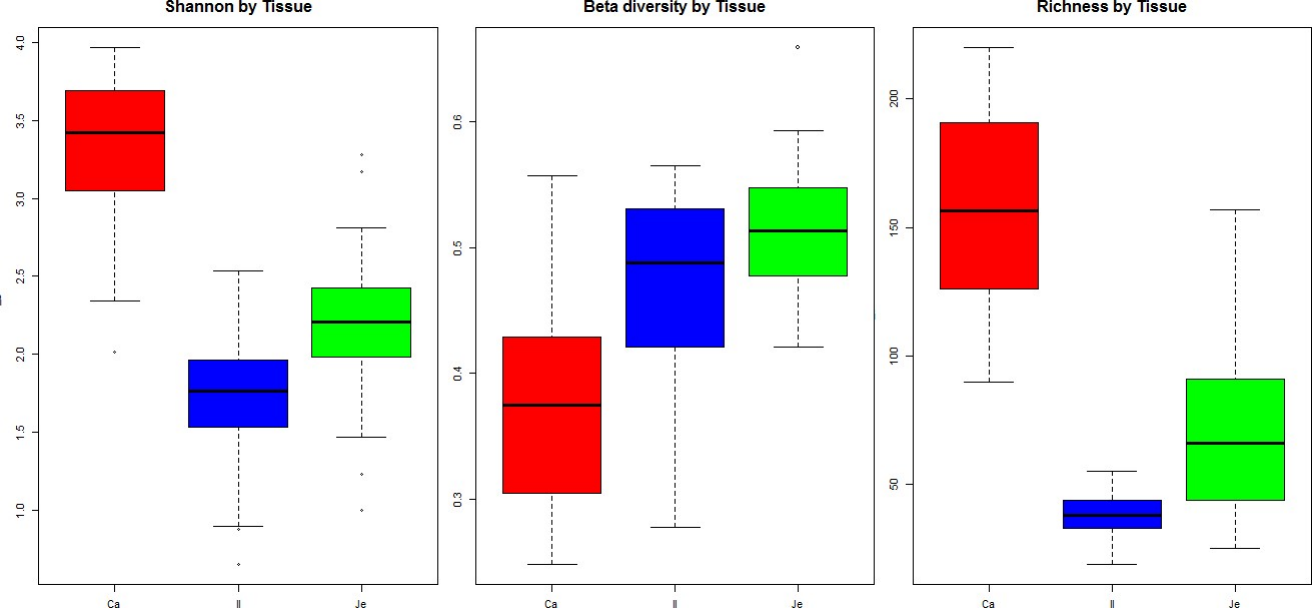
Alpha-diversity, beta-diversity and richness of microbiota samples from the caeca (Ca), ileum (Il) and jejunum (Je) of the subset of 39 chickens chosen according to their AMEn.

Fig 2 shows the alpha-diversity, beta-diversity and richness in ileum, jejunum and caeca. As expected from similar studies, richness was much higher in caeca than in jejunum and ileum, with 250 OTUs on average, compared with 50 OTUs for the ileal segment, and 150 OTUs for the jejunum segments (*P* = 1.44 10^−14^). Similarly, alpha-diversity was higher in caeca compared to the two other segments, while beta-diversity was the lowest in caecal samples, intermediate in ileum samples and high in jejunum samples.

#### Compostion at the phylum level

*Firmicutes* was the dominant phylum in the three intestinal segments studied, and was statistically more abundant in ileum (average abundance 85%) than in jejunum and caeca (average abundances 66.6 and 74.0% respectively, p-adj = 1.1 10^−5^) (Table 1). In a lesser extent, two other phyla were found with abundances above 2% in the caeca: *Bacteroidetes* (15%) and *Actinobacteria* (2.4%). Those same phyla and *Proteobacteria* were found in jejunum and ileum, although with a different ranking according to their relative abundances: first *Proteobacteria* (resp. 15.3% and 9.7%), followed by *Actinobacteria* (resp. 7.0% and 4.1%) and *Bacteroidetes* (resp. 4.7% and 0.5%). *Proteobacteria* and *Actinobacteria* were thus both significantly more abundant in ileum and jejunum than in caeca, while *Bacteroidetes* was significantly more abundant in caeca than in jejunum.

**Table 1:** Relative taxonomic composition (in percentages) of microbiota samples at different taxonomic ranks, according to their intestinal segment of origin.

Phylum	Caeca	Ileum	Jejunum	p-value	FDR p-adj
*Firmicutes*	74.0 ^b^	84.4 ^a^	66.6 ^b^	6.5 10^−6^	1.1 10^−5^
*Bacteroidetes*	15.1 ^a^	0.5 ^c^	4.7 ^b^	5.8 10^−12^	4.1 10^−11^
*Actinobacteria*	2.4 ^b^	4.1 ^a^^b^	7.0 ^a^	4.5 10^−3^	5.3 10^−3^
*Proteobacteria*	1.5 ^b^	9.7 ^a^	15.3 ^a^	3.8 10^−7^	8.8 10^−7^
*Cyanobacteria*	0.02 ^c^	0.2 ^b^	3.8 ^a^	1.1 10^−8^	3.7 10^−8^
**Family**	Caeca	Ileum	Jejunum	p-value	FDR p-adj
*Ruminococcaceae*	56.8 ^a^	3.2 ^b^	7.7 ^b^	6.5 10^−46^	3.3 10^−44^
*Rikenellaceae*	13.1 ^a^	0.2 ^b^	2.3 ^b^	1.5 10^−13^	1.9 10^−12^
*Lachnospiraceae*	8.1 ^a^	3.9 ^a^^b^	1.2 ^b^	0.003	0.01
*Lactobacillaceae*	2.1 ^b^	64.6 ^a^	50.8 ^a^	1.8 10^−25^	4.7 10^−24^
*Enterobacteriaceae*	0.9 ^b^	3.9 ^a^	0.4 ^b^	2 10^−4^	0.001
*Clostridiaceae*	0.08 ^b^	8.1 ^a^	1.0 ^b^	5.1 10^−8^	4.4 10^−7^
*Peptostreptococcaceae*	0.1	4.1	4.9	0.04	0.1
*Microbacteriaceae*	0.3 ^b^	2.3 ^a^	3.1 ^a^	4.8 10^−6^	3.5 10^−5^
*Xanthomonadaceae*	0.1 ^b^	1.7 ^a^	4.1 ^a^	0.002	0.007
*Nocardiaceae*	0.1 ^b^	1.2 ^a^	2.0 ^a^	0.008	0.02
*Comamonadaceae*	0.07 ^b^	1.1 ^a^	3.2 ^a^	0.007	0.02
*Caulobacteraceae*	0.1 ^b^	0.7 ^a^	2.6 ^a^	9.2 10^−4^	0.004
** Genus**	Caeca	Ileum	Jejunum	p-value	FDR p-adj
*Faecalibacterium*	35.1 ^a^	2.4 ^b^	4.9 ^b^	3.9 10^−29^	1.1 10^−27^
*Oscillospira*	9.8 ^a^	0.3 ^b^	1.1 ^b^	6.7 10^−42^	3.6 10^−40^
*Butyricicoccus*	3.1 ^a^	0.1 ^b^	0.3 ^b^	8.4 10^−14^	6.5 10^−13^
*Ruminococcus*	2.2 ^a^	0.08 ^b^	0.18 ^b^	2.5 10^−25^	3.4 10^−24^
*Lactobacillus*	2.1 ^b^	64.6 ^a^	50.8 ^a^	1.8 10^−25^	3.3 10^−24^
*Candidatus Arthromitus*	0.06 ^b^	7.9 ^a^	0.9 ^b^	8.4 10^−8^	3.8 10^−7^
*Clostridium*	0.08	3.3	0.9	0.07	0.13
*Microbacterium*	0.3 ^b^	2.2 ^a^	4.0 ^a^	9.5 10^−6^	3.4 10^−5^
*Stenotrophomonas*	0.1 ^b^	1.7 ^a^	4.0 ^a^	0.002	0.004
*Rhodococcus*	0.1 ^b^	1.2 ^a^	2.0 ^a^	0.008	0.02

Only taxons with percentages higher than 2% in at least one intestinal segment are showed. For each taxon, a statistical test was conducted to assess the significance of differences of abundance between segments of origin; p-values were corrected for controlling the false discovery rate (FDR)

a, b, c: groups significantly different for each taxon; no letter: no significant difference between intestinal segments.

#### Composition at the family level

Overall, we detected 53 families and differences between caeca and the two upper segments (ileum and jejunum) were even more marked than at the phylum level (Table 1). *Ruminococcaceae* and *Lactobacillaceae* were present with abundances above 50% in at least one segment, with *Ruminococcaceae* more abundant in caeca (56.8%) than in ileum (3.2%) and jejunum (7.7%) (FDR *P =* value = 3.3.10^−44^), and *Lactobacillaceae* more abundant in ileum (64.6%) and jejunum (50.8%) than in caeca (2.1%) (FDR *P =* 4.7.10^−24^). To a lesser extent, *Rikenellaceae*, *Lachnospiraceae*, and *Clostridiaceae* were present with abundances above 5% in at least one segment. *Rikenellaceae* and *Lachnospiraceae* more abundant in caeca (13.1% and 8.1% resp.) than in ileum (0.2% and 3.9% resp.) and jejunum (2.3% and 1.2% resp.; FDR *P =* 1.9.10−^12^ and 0.01). *Clostridiaceae* was more abundant in ileum (8.1%) than in caeca (0.08%) and jejunum (1.0%) (FDR *P =* 4.4.10^−7^).

#### Composition at the genus level

**Fig 3:**
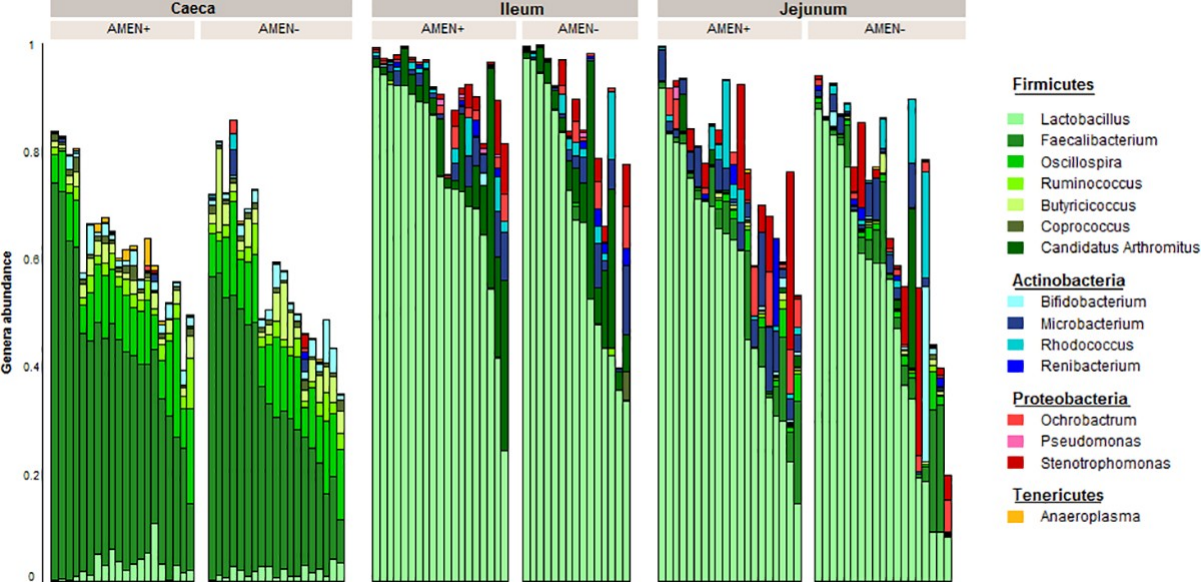
OTU composition of chicken microbiota samples at the genus level according to their intestinal segment of origin (caeca, ileum, or jejunum) and to the digestibility level of the animals (AMEn+ or AMEn-).

We detected 55 genus present in the samples. *Lactobacillus* and *Faecalibacterium* were present with abundances above 30% in at least one segment, with *Faecalibacterium* more abundant in caeca (35.1%) than in ileum (2.4%) and jejunum (4.9%; FDR *P =* 1.1.10^−27^), and *Lactobacillus* being more abundant in ileum (64.6%) and jejunum (50.8%) than in caeca (2.1%; FDR *P =* 3.3.10^−24^) (Fig 3). In a lesser extent, *Oscillospira*, *Butyricicoccus*, *Candidatus Arthromitus*, *Clostridium*, *Microbacterium*, and *Stenotrophomonas* were present with abundances above 3% in at least one segment. *Oscillospira* and *Butyricicoccus* were more abundant in caeca (9.8% and 3.1% resp.) than in ileum (0.3% and 0.1% resp.) and jejunum (1.1% and 0.3% resp.; FDR *P =* 3.6.10^−40^ and 6.5.10^−13^). *Candidatus Arthromitus* and *Clostridium* were more abundant in ileum (7.9% and 3.3% resp.) than in caeca (0.06% and 0.08% resp.) and jejunum (0.9% and 0.9% resp.; FDR *P =* 3.8.10^−7^ and 0.13 resp.). *Microbacterium* and *Stenotrophomonas* were more abundant in jejunum (4.0% and 4.0% resp.) than in caeca (0.3% and 0.1% resp.) and ileum (2.2% and 1.7% resp.; FDR *P =* 3.4.10^−5^ and 0.004).

### Differences in OTU abundances between AMEn+ and AMEn- animals

The digestibility group was not significantly associated OTU richness in caeca (*P* = 0.23), ileum, (*P* = 0.10) or jejunum (*P* = 0.85). However, significant differences in specific OTU abundances occurred, especially in caeca, and in a lesser extent in jejunum and ileum. For further discussion, we decided to consider only OTUs present in at least half of the samples from AMEn- or AMEn+ group; below this threshold, the OTUs had very low counts in a few samples (S5 Table).

#### Caeca

At the OTU level (Table 2), after excluding OTUs that were lacking in at least half of the samples, 27 OTUs were significantly differentially abundant in caeca: 11 OTUs were more abundant in AMEN+ vs 16 in AMEn- animals. In AMEn+ samples, the most abundant OTUs included bacteria from two different orders: *Bacteroidales* and *Clostridiales*, while two OTUs had no taxonomic annotation. *Bacteroidales* bacteria belonged to the *Rikenellaceae* family, while *Clostridiales* bacteria either belonged to the *Ruminococcaceae* family or could not be assigned at the family level. At the genus level (Table 3), *Anaeroplasma* and *Clostridia* were more abundant in AMEn+ samples. At the family level (Table 4), the families *Anaeroplasmataceae* and *Desulfovibrionaceae* are more abundant in AMEN+ samples.

**Table 2:** List of OTUs significantly more abundant in AMEn+ or AMEn- caecal samples, with OTUs compared at the species level.

Digestibility group	OTU	Phylum	Class	Order	Family	Genus	Species	pvalues	adjPvalues
AMEN^+^	351292	*Bacteroidetes*	*Bacteroidia*	*Bacteroidales*	*Rikenellaceae*	*Alistipes*	*Massiliensis*	0.000	0.002
AMEN^+^	185420	-*	-	-	-	nd**	Nd	0.000	0.003
AMEN^+^	4476780	-	-	-	-	Nd	Nd	0.000	0.009
AMEN^+^	758482	*Firmicutes*	*Clostridia*	*Clostridiales*	*Ruminococcaceae*	Nd	Nd	0.000	0.005
AMEN^+^	denovo135220	-	-	-	-	*Oscillospira*	Nd	0.001	0.013
AMEN^+^	593709	-	-	-	-	*Ruminococcus*	Nd	0.000	0.000
AMEN^+^	546958	-	-	-	-	Nd	Nd	0.000	0.010
AMEN^+^	155362	-	-	-	*Lachnospiraceae*	*Clostridium*	*Piliforme*	0.002	0.026
AMEN^+^	237063	-	-	-	nd	Nd	Nd	0.004	0.046
AMEN^+^	197427	-	-	-	nd	Nd	Nd	0,000	0.008
AMEN^+^	562369	-	-	-	nd	Nd	Nd	0.002	0.028
AMEN-	443620	*Firmicutes*	*Clostridia*	*Clostridiales*	*Ruminococcaceae*	*Oscillospira*	Nd	0.003	0.038
AMEN-	131039	-	-	-	-	-	Nd	0.001	0.019
AMEN-	839964	-	-	-	-	-	Nd	0.003	0.038
AMEN-	197874	-	-	-	-	*Butyricicoccus*	*pullicaecorum*	0.003	0.038
AMEN-	193240	-	-	-	-	-	-	0.002	0.029
AMEN-	738351	-	-	-	-	-	-	0.002	0.035
AMEN-	1132942	-	-	-	-	-	-	0.001	0.023
AMEN-	195415	-	-	-	-	*Ruminococcus*	Nd	0.003	0.038
AMEN-	278098	-	-	-	-	-	-	-	-
AMEN-	denovo1280	-	-	-	-	*Anaerotruncus*	Nd	0.000	0.005
AMEN-	198009	-	-	-	-	Nd	Nd	0.002	0.035
AMEN-	166637	-	-	-	*Lachnospiraceae*	*Dorea*	Nd	0.000	0.008
AMEN-	199276	-	-	-	-	*Coprococcus*	Nd	0.000	0.004
AMEN-	581014	-	-	-	-	Nd	Nd	0.001	0.015
AMEN-	523064	-	-	-	-	Nd	Nd	0.001	0.019
AMEN-	772384	-	-	-	-	Nd	Nd	0.000	0.004

** “-“: taxonomy identical as the one mentioned in the previous line

*“nd”: undetermined taxon

**Table 3:** List of OTUs significantly more abundant in AMEn+ or AMEn- caecal samples, at the genus level.

Digestibility group	Phylum	Class	Order	Family	Genus	pvalues	adjPvalues
AMEN^+^	*Tenericutes*	*Mollicutes*	*Anaeroplasmatales*	*Anaeroplasmataceae*	*Anaeroplasma*	0.008	0.032
AMEN^+^	*Firmicutes*	*Clostridia*	*Clostridiales*	*Lachnospiraceae*	*Clostridium*	0.009	0.034
AMEN^+^	*Proteobacteria*	*Deltaproteobacteria*	*Desulfovibrionales*	*Desulfovibrionaceae*	Nd	0.002	0.008
AMEN-	*Proteobacteria*	*Gammaproteobacteria*	*Enterobacteriales*	*Enterobacteriaceae*	*Escherichia*	0.014	0.05

**Table 4:** List of OTUs significantly more abundant in AMEN+ caecal samples, at the family level. No significantly differentially abundant OTU was identified at the family level in AMEN- samples.

Digestibility group	Phylum	Class	Order	Family	Pvalues	adjPvalues
AMEN^+^	*Tenericutes*	*Mollicutes*	*Anaeroplasmatales*	*Anaeroplasmataceae*	0.005	0.022
AMEN^+^	*Proteobacteria*	*Deltaproteobacteria*	*Desulfovibrionales*	*Desulfovibrionaceae*	0.001	0.004

In AMEn- samples, all OTUs had a taxonomic description at the family level (Table 2), and most of them at the Genus level (Table 3). All OTUs more abundant in AMEn- animals belonged to the *Clostridia* order, with representatives from two families: *Ruminococcaceae* and *Lachnospiraceae*. The four OTUs annotated at the species level (Table 2) belonged to the species *Butyricicoccus pullicaecorum*. At the genus level (Table 3), the genus *Escherichia* was more abundant in AMEn- samples.

Among the sets of OTUs significantly differentially abundant between AMEn groups, ten were also present in the functional predictions described below. More specifically, OTU 593709 from the *Ruminococcus* genus, OTU 758482 from the *Ruminococcaceae* family, OTU 546958 from the *Ruminococcaceae* family, and OTU 582181 from the *Mollicutes* class are also observed among the main OTUs contributing to the set of functions predicted to be more abundant in caecal contents of AMEn+ animals. Conversely, the main OTUs contributing to the set of functions predicted to be more abundant in AMEn- caecal contents were OTU 523064 from the *Lachnospiraceae* family, OTUs 738351, 197874 and 1132942 from the *Butyricicoccus pullicaecorum* species, and OTUs 443620 and 839964 from the *Oscillospora* genus.

#### Jejunum

In jejunum microbiota samples, 66 OTUs were differentially abundant between AMEn+ and AMEn- samples at the species level (Table 5). Nevertheless, most of them were present in very few samples: only two of them were identified in more than half of the samples. One OTU (OTU 4402645) was more abundant in AMEn+, the other one (OTU 183932) belonged to the *Butyricicoccus pullicaecorum* species and was more abundant in AMEN- samples (Table 5). At higher taxonomic ranks, the Blautia genus was more abundant in AMEn- samples (adj pvalue = 0.0036).

**Table 5:** List of OTUs significantly more abundant in AMEN+ or AMEN- jejunal samples, at the species and genus levels.

Species									
Digestibility	OTU	Phylum	Class	Order	Family	Genus	Species	Pvalues	adjPvalues
AMEN^+^	4402645	*Firmicutes*	*Clostridia*	*Clostridiales*	Nd	nd	Nd	0.009	0.049
AMEN^-^	183932	*Firmicutes*	*Clostridia*	*Clostridiales*	*Ruminococcaceae*	*Butyricicoccus*	*Pullicaecorum*	0.002	0.018
**Genus**									
**Digestibility**	**OTU**	**Phylum**	**Class**	**Order**	**Family**	**Genus**	**Species**	**Pvalues**	**adjPvalues**
AMEN^-^	--	*Firmicutes*	*Clostridia*	*Clostridiales*	*Lachnospiraceae*	*Blautia*	--	0.000	0.000

#### Ileum

In ileum samples, 38 OTUs were differentially abundant at the species level between AMEn+ and AMEn- samples. Nevertheless, most of them were present in very few samples: only one of them was identified in more than half of the samples (Table 6): a *de novo* OTU more abundant in AMEn+ animals (annotated at the order level as a *Bacteroidales bacterium*).

**Table 6:** OTU significantly more abundant in AMEn+ or AMEn- ileal samples, at the species level.

Digestibility	OTU	Phylum	Class	Order	Family	Genus	Species	pvalues	adjPvalues
AMEN^+^	denovo80881	*Bacteroidetes*	*Bacteroidia*	*Bacteroidales*	*Barnesiellaceae*	nd	Nd	0.000	0.002

### Functional prediction of metabolic differences between AMEn+ and AMEn- animals

#### Caeca

The mean NSTI of the caecal communities analyzed was 0.1 on a 0 to 1 scale. Among the 5,286 functions predicted using PICRUSt, 50 were statistically more abundant in the AMEn+ group than in the AMEn- group (Deseq2 Analysis). Among these 50 functions, only 17 had a known pathway identifier (S2 Table).

**Fig 4:**
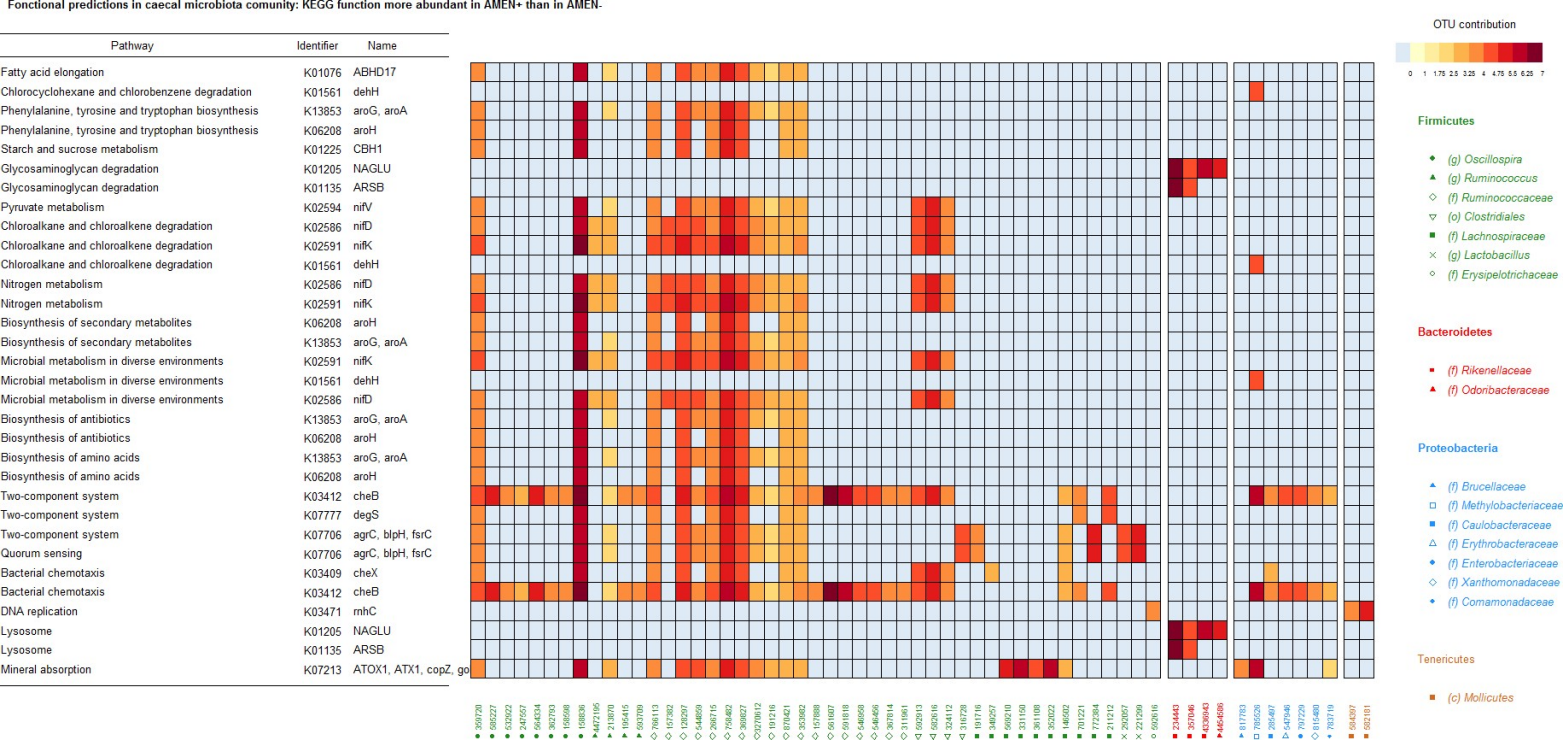
Functional predictions inferred from the OTU composition of caecal samples from AMEn+ animals (high digestive efficiency).

**Fig 5:**
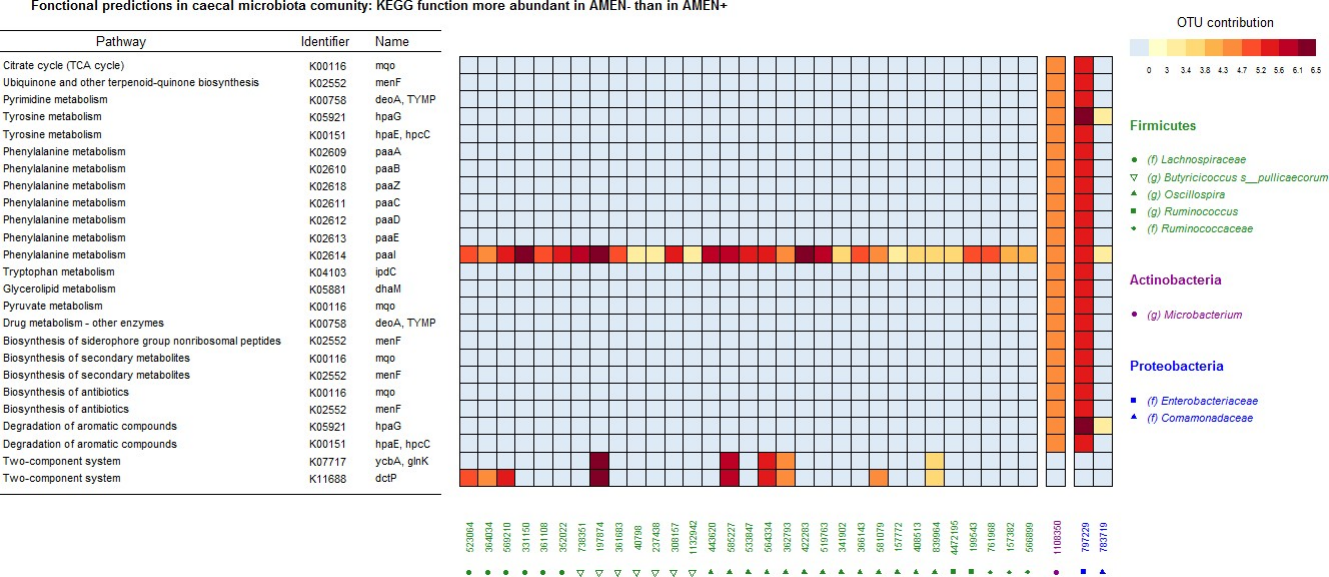
Functional predictions inferred from the OTU composition of caecal samples from AMEn- animals (low digestive efficiency).

We first paid more attention to functions related to lipid, amino-acids and glucose metabolism, which were more abundant in AMEn+ samples. Regarding lipid metabolism, we observed the K01076 function related to fatty acid elongation and coding for a hydrolase enzyme acting on palmitoyl substrate. Regarding amino acids metabolism, we observed the K06208 and K13853 functions related to phenylalanine and tyrosine metabolism. At last, for glucose metabolism we observed two functions: the K01225 function related to starch and sucrose metabolism due to a glycosylase enzyme acting on β1-4-DM glucosidic linkages from cellulose and cellotetraose, and the function K02594 from pyruvate metabolism linked subsequently to lysine biosynthesis. When considering the putative contribution of specific OTUs to these functions (Figs 4 and 5), it appears that this set of functions was considerably contributed by the OTU 158836 from the *Oscillospora* genus and to a lesser extend to OTUs 758482, 369827, 128297, and 266715 from the *Ruminococcaceae* family. Besides, glycosaminoglycan degradation was predicted to be more abundant in AMEn+ than in AMEn- through the K01205 and K01135 functions displayed by Bacteroidetes, mainly due to 3 OTUs (OTU 234443, OTU 357046, and OTU 4336943) from the *Rikenellaceae* family and to a lesser to the OTU 4454586 from the *Odoribacteraceae* family.

Other functions identified were related to DNA replication, bacterial chemotaxis, quorum sensing, and the two-component system and were predicted to be more abundant in AMEn+ than in AMEn- (S2 Table). The latter predicted functions were essentially due to: OTU 582181 and 584397 (*Mollicutes* class, phylum *Tenericutes*) for DNA replication; OTU 158836 (*Oscillospora* genus), OTU 561607, OTU 591818 (*Ruminococcaceae* family) and OTU 785526 (*Methylobacteriaceae* family) for bacterial chemotaxis; OTU 158836 (*Oscillospora* genus), OTU 758482 (*Ruminococcaceae* family), OTU 772384 (*Lachnospiraceae* family) and OTU 221299 (*Lactobacillus* genus) for the quorum sensing function K07706 (Fig 4).

Twenty-three functions were statistically more abundant in the AMEn- group than in the AMEn+ group. Among these functions, only 17 had a known pathway and KEGG identifier (S2 Table). Except for KEGG genes K07717 and K11688 from the two-component system pathway, only two OTUs (OTU 797229 from the *Enterobacteriaceae* genus and in a lesser extend OTU 1108350 from the *Microbacterium* genus) contributed predominantly to this set of more abundant functions. The KEGG gene K05881, involved in glycerolipids metabolism, was the only function involving lipids. Most of the functions related to amino-acids metabolism engaged phenylalanine, only two engaged tyrosine, and one tryptophan.

#### Ileum

**Fig 6:**
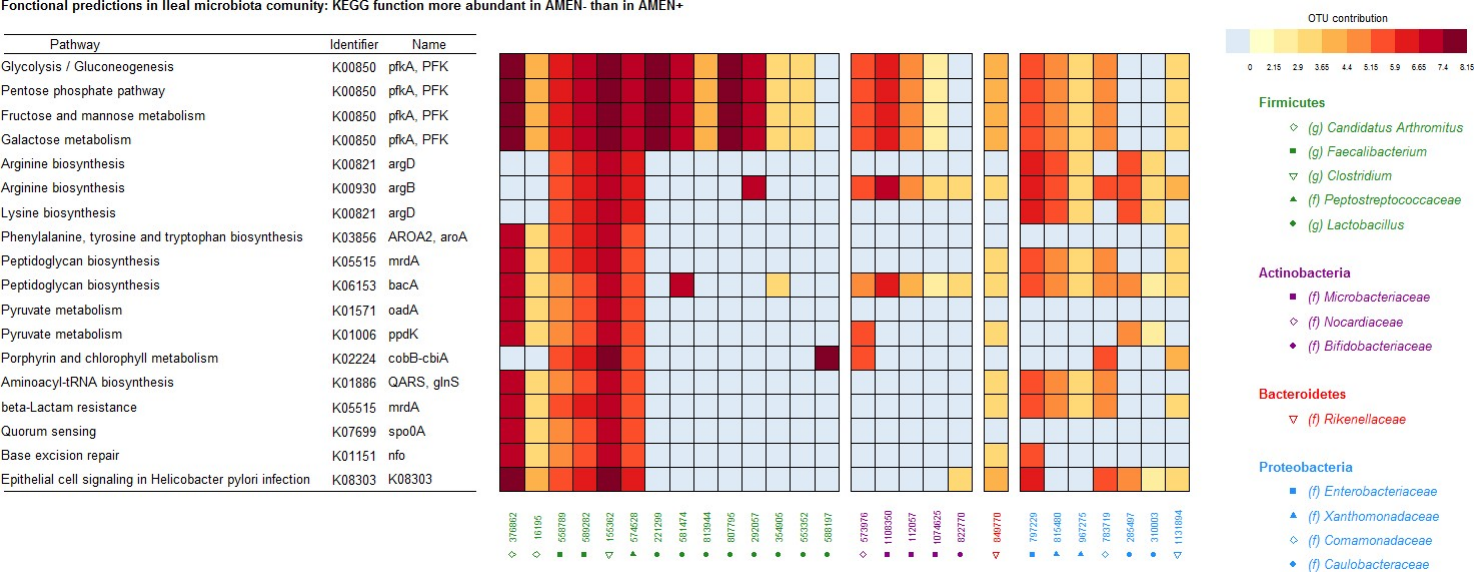
Functional predictions inferred from the OTU composition of ileal samples from AMEn- animals (low digestive efficiency).

The mean NSTI from ileum microbial communities was 0.05. Among the 5,420 predicted functions in ileum, 38 were enriched in AMEn- compared to AMEn+, including 13 with a known pathway and KEGG identifier (S2 Table). Regarding glucose metabolism, the KEGG function K00850 contributing to glycolysis/gluconeogenesis, pentose phosphate pathway, fructose and mannose metabolism, and galactose metabolism due to 6-phosphofructokinase 1 enzyme was predicted to be more abundant in AMEn-. These differentially abundant functions were essentially due to OTU 376862 (*Candidatus Arthromitus* genus), OTU 155362 (*Clostridium* genus), OTUs 221299 and 807795 (*Lactobacillus* genus), and to a lesser extend to OTU 558789 and 589282 (*Faecalibacterium* genus), OTU 574528 (*Peptostreptococcaceae* family), OTU 581474 and 292057 (*Lactobacillus* genus), and to OTU 1108350 (*Microbacteriaceae* family) (Fig 6). Concerning amino-acids metabolism, the K00821 and K00930 functions related to arginine and lysine biosynthesis pathway due to acetylornithine/N-succinyldiaminopimelate aminotransferase and acetylglutamate kinase respectively were predicted to be more abundant in AMEn- (S2 Table). These predicted functions were essentially due to OTU 558789, OTU 589282, OTU 155362 (Clostridium genus), OTU 574528 (*Peptostreptococcaceae* family), OTU 292057 (*Lactobacillus* genus), OTU 1108350 (*Microbacteriaceae* family), and OTU 797229 (*Enterobacteriaceae*) family (Fig 6).

#### Jejunum

Only 3 functions were predicted to be differentially abundant between AMEn+ and AMEn- samples. The KEGG function K10027 was the only one with a pathway available with the Clusterprofiler package. However, this function assigned as a phytoene desaturase is not directly involved in nutrient metabolism.

## Discussion

In previous studies, Mignon-Grasteau et al. [2] first demonstrated that the digestibility of animals fed a difficult to digest diet (wheat with a high viscosity) is highly variable, and that part of this phenotypic variability is genetically heritable. A successful genetic selection experiment from this commercial broiler population then led to two chicken lines harboring contrasted values of digestibility: either low (D-) or high (D+). Beyond anatomical and physiological traits, Mignon-Grasteau et al. [6] demonstrated that animals derived from these lines harbor differential abundances in specific bacteria species or groups. Here we confirm and extend these results by using a more exhaustive method: a 16S rRNA gene sequencing approach, i.e. without *a priori* as to the bacterial species potentially present. We confirm that the divergent genetic selection led for digestive efficiency had an impact on the whole gut microbiota composition and not only on specifically targeted bacteria groups.

### Exploratory analysis

Gut microbiota composition, in chicken as in other animal species, is highly dynamic and varies according to many different parameters describing the animal as well as its immediate environment and the feed it receives. Known parameters include both intrinsic and extrinsic factors: respectively age, breed, sex, and feed, housing, hygiene, medication, temperature, litter or location. In addition, the composition of gut microbiota changes along the intestinal tract, which we confirm here through the comparison of caeca, jejunum and ileum microbiotas. Birds in our experiment received identical feed and we raised them in strictly the same conditions, so that many parameters were fixed (age, feed, prophylaxis, breeding environment). In these highly controlled conditions, the main driver of gut microbiota composition was the intestinal segment of origin, which could be expected given the strong differences already reported in bacterial composition along the digestive tract in many studies [23]. This observation is consistent with previous results showing that the segment of origin was also the main driver for host intestinal transcriptome between D+ and D- birds bred in similar conditions [24]. The differences observed here between jejunal, ileal and caecal composition, with ileal, jejunal and caecal microbiota forming distinct clusters according to our NMDS analysis, are in accordance with similar, published results. *Lactobacillus* is thus the predominant genus in ileum and jejunum, as was previously observed [6], and *Faecalibacterium* in caeca [25–27]. Caeca harbour a much greater taxonomic diversity and richness and are dominated by bacteria from the Firmicutes phylum, followed by Bacteroidetes, Actinobacteria and Proteobacteria [23].

### Influence of the digestibility level on the gut microbiota

The second parameter of influence on microbiota composition in our experiment was the digestibility level. Although it had no effect on overall alpha- and beta-diversity and richness in the intestinal segments studied, it was associated with differences in OTU abundances in ileum, jejunum and caeca. In upper segments of the digestive tract, the differences in abundances between OTUs are linked to a few individuals. In contrast, most of the OTUs in caecal samples were present in more than half of the samples. The strongest association between digestibility group and OTU composition differences therefore occurred in the caeca. It reinforces similar observations of association between feed efficiency and gut microbiota composition in commercial broilers [27] and most probably reflects the functional involvement of the caecal microbiota in the digestive efficiency of its host. While nutrient absorption mainly occurs in the upper part of the digestive tract, the fermentation of complex polysaccharides such as those found in wheat mainly occurs in caeca. The longer retention time observed in the caeca of D+ animals [5] could allow bacteria with a slower growth to proliferate. The pH of intestinal contents differs between D+ and D- animals [28]. Moreover, feed particles in D+ are smaller and thus more accessible to bacteria, due to a stronger mechanic grinding of feed in the bigger gizzards of D+ animals. At last, caeca and caecal contents of D+ animals are heavier [28]. These differences affect the caecal biotope from which bacteria develop and it seems likely that these modifications affect the developing microbiota in chicks. Animals in our experiment are the progeny of 8 generations of [D+ x D-] intercross, therefore genes controlling digestive efficiency are segregating and animals harbor different combinations of alleles at genes controlling variations in digestibility. Arguably, differences in digestibility levels between birds might be explained in part by the colonization of the digestive tract by distinct sets of bacteria from the immediate environment at hatch, possessing distinct abilities to digest the feed ingested and thus leading to contrasted feed efficiencies. Some studies indeed formulate this hypothesis to explain the high individual variability of gut microbiota composition of birds they observe even in highly controlled conditions [29]. It is likely, though, given the strong divergence of the two parental lines [11,30], that most of the variation observed between extreme animals is caused by genetic differences. This is strengthened by the high heritability of digestibility [2] and by the identification of several QTL (quantitative trait loci) controlling digestibility in a [D+ x D-] F2 progeny [31]. Genetic selection might thus affect the gut microbiota through a modification of its biotope, or possibly through other mechanisms not yet identified in this precise case, such as changes in the host-microbiota crosstalk leading to a selective colonization of the digestive tract [32]. Differences in the histology of the intestinal epithelium have been observed between D+ and D- birds [33,34], which might affect this crosstalk. The identification of the genes controlling changes in the gut microbiota could help us at identifying the molecular pathways involved on the host side. Among the candidate genes identified through QTL analyses [31] or transcriptomics studies [24] led on D+/D- animals, several are involved in host immunity and might regulate the host-microbiota crosstalk. To complete these studies, identifying the genes controlling the abundances of differentially abundant OTUs between AMEN+ and AMEN- samples would be of high interest, to confirm and fine-map the QTLs identified and enlarge the panel of putative candidate genes controlling gut microbiota composition.

### Identification of differentially abundant OTUs

Some of the differentially abundant OTUs we identified were also identified in previous, comparable studies. In our previous study of animals derived from a F2 [D+ x D-] cross [6], *Escherichia coli* and *Lactobacillus salivarius* were more abundant in animals with the highest FCR (correlated with lowest digestibility). Here we partially confirm these results, since the genus *Escherichia* is actually more abundant in AMEn- animals, while we did not observe difference in abundance of neither *Lactobacillus salivarius* nor *Lactobacillus*. Differences in experimental design and phenotype measured are the likely causes of this apparent discrepancy: although fed with similar diets, animals belong to different generations and in the first experiment, contrasted groups were defined according to their FCR and not to their AMEn as in the present study. Although highly correlated, these parameters are not identical. Whereas FCR describes the ability to convert feed into body weight, AMEn describes the ability to extract energy from feed. The microbiota of high/low FCR animals might be different from that of high/low AMEn animals. Thus, another study already reported the very reduced overlap between OTU associated with AMEn and OTU associated with FCR [35,36]. *Lactobacillus* as a taxon has been identified for its association with a lower residual feed intake [37], but not with AMEn to our knowledge, in line with the absence of association observed in this study. It might be involved in the conversion of feed to body weight, probably through the digestion of lipids via the hydrolysis of biliary salts mainly observed in the upper part of the digestive tract, but not in the extraction of energy from feed. *Lactobacillus* species have been associated with a better fed efficiency in several studies. Nevertheless, Stanley et al. [36] observed that some other *Lactobacillus* species can be associated with a weaker feed efficiency in the same experiment, so that a great caution is needed before generalizing results. Associations seem to be species-specific and can even change according to the experiment [36]. At last, *E*. *coli* is considered as a potentially pathogenic and detrimental species, so that its identification in animals with low digestibility might mean that these animals, in addition to a poor ability to extract energy from feed, also have to cope with a potentially pathogenic microbiota which could lead to diseases under unfavorable conditions.

Beside our previous study, other studies looked for associations between variations for different traits characterizing feed efficiency (FCR, RFI, AMEn) and variations in the composition of the digestive microbiota. Only one of them though used AMEn as a way of valuating performances [35]. For instance, while we identified *Alistipes massiliensis* for its association with a higher digestibility, an OTU from the *Alistipes* genus was found to be associated with a better feed efficiency estimated through FCR [27]. Another study showed that OTUs from the *Clostridiales* family were the OTUs the most often associated with a low FCR [36], which seems in line with our results since most of the OTUs more abundant in the AMEn+ animals are from this family. Nevertheless, all of the OTU associated with AMEn+ also belong to this family, so that we have to conclude that it is not the family per se which is associated with poorer/ higher performances, but rather species and maybe even strains of bacteria, similarly to the observation made by Stanley et al [36] about *Lactobacillus*.

Interestingly, among the OTUs more abundant in the caeca of AMEn- animals, four belong to the species *Butyricicoccus pullicaecaorum*. Furthermore, another OTU of this species was identified in the jejunum of AMEn- animals. This bacterium produces butyrate, a short-chain-fatty-acid resulting from the degradation of complex carbohydrates in anaerobic conditions, otherwise known for its anti-inflammatory effects [38]. Broilers fed with this bacterium showed a decreased feed conversion ratio (FCR) and a reduced quantity of potential pathogenic bacteria [39]. In addition, birds challenged for necrotic enteritis and fed this bacterium displayed a reduced quantity of necrotic lesions [40]. This potential probiotic bacterium thus provides positive effects on both a productivity-related trait and on health-related traits when directly fed to animals. In our study though, the animals with the highest digestive efficiency (i.e. lowest FCR, given the negative correlation occurring between FCR and digestibility) carried *B*. *pullicaecorum* in the lowest quantity. It does not imply that the gut health of D+ animals, otherwise not assessed in this study, is poorer. Animals in our study were not fed with this bacterium and thus harbor only the naturally occurring *B*. *pullicaecorum*. Besides, other butyrate-producing bacteria might be present such a *Faecalibacterium* that was detected in our study. It would be of high interest to know the actual quantity of butyrate and other SFCA produced by the caecal microbiota in the divergent D+/D- lines, to know whether the differential abundance of *B*. *pullicaecorum* OTU actually reflects a more general trend toward an increased production of SCFA in AMEn- birds. Furthermore, assessing health-related traits in D+/D- birds in relation to the gut microbiota composition would be of interest to check the putative effect on health of the selection on high/ low digestibility. We thus previously showed that D+ animals are less susceptible to an avian pathogenic *E*. *coli* (APEC) strain [40].

### Functional analysis

Approaches relying on the sequencing of the 16S rRNA gene do not allow the direct identification of bacterial genes. Nevertheless, tools such as PICRUSt are available, which are able to infer putative genes from genomic information collected in databases on the taxons identified [22]. These tools rely on the relevance of the initial OTU annotation, as well as on the quality and quantity of data available on the related bacterial species. Furthermore, databases contain information collected mainly from human and Mammal samples and thus might not well represent samples from chicken, which we confirm here with the identification of low NSTI levels. Therefore, results must be interpreted with caution. With 73 predicted functions differing in their abundances in caeca between AMEn+ and AMEn- animals, versus 38 in ileum, and only 3 in jejunum, caeca is the intestinal segment the more impacted by the differences in digestive efficiency at the functional level. Caecal microbiota realizes important functions: nitrogen recycling by breakdown of uric acid [41], B vitamin production [42], essential amino acids production and non-starch polysaccharides (NSPs) digestion. Cereals, which are the main feedstuffs in poultry diets have various levels of NSPs, which is known to affect the microbiota composition. For example wheat, which was used in the present study as the main cereal source, contains more NSP than maize. Host enzymes do not digest NSP well; their digestion requires the action of bacteria equipped with enzymes such as glycosyl hydrolases. A higher proportion of NSP through a diet containing more wheat, for instance, is known to trigger an increased retention time of feed and a proliferation of slow-growing bacteria able to degrade NSP. The higher abundance of the K01225 function related to starch and sucrose metabolism due to cellulose 1,4-beta-cellobiosidase observed in AMEn+ animals suggests that the fermentative process of NSPs is more intense in AMEn+ animals. Furthermore, in the present study, a genus from the *Desulfovibrionaceae* family was significantly associated with AMEn+ in caeca. At the functional level, *Desulfibrio spp* have already been reported to be potential hydrogen consumers able to oxidize H_2_ produced during NSP fermentation into short-chain volatile fatty acids (SCFA) [43]. Consequently, the greater abundance of *Desulfovibrionaceae* in AMEn+ may suggest that higher NSP fermentation and SCFA production are present in AMEn+ animals, which could contribute to the greater digestive performances observed in these animals. This potential involvement of OTU from the *Desulfovibrionaceae* family in the fiber fermentative process is consistent with previous observations. The abundances of OTU from the *Desulfibrio* genera were also higher in the most performant broilers (high body weight, low FCR) fed with the probiotic *Butyricoccus pullicaecorum* [39].

Those functional predictions provide some interesting investigative tracks about the functional involvement of the gut microbiota in the digestive processes. They tend to confirm that the caecal ecosystem of AMEn+ birds, as a direct or indirect consequence of genetic selection, possesses a higher aptitude at digesting NSPs, which are present in a higher proportion in wheat. Such predictions remain to be validated, though, since they identify only putative functions through *in silico* approaches, with many biases. True functional approaches through whole metagenome sequencing coupled with metabolomics, although expensive, could allow a more precise and relevant analysis of the functions involved in the better digestibility of AMEn+ birds.

## Conclusion

We confirmed that the digestive microbiota of AMEn+ and AMEn- birds selected within a [D+ x D-] F8 progeny differ mainly in the caeca, with significant differences in the abundance of several OTUs, some of which might contribute to the degradation of NSPs and the production of SCFA. Identifying the host genes controlling the abundance of these OTUs might give us further insights as to the host pathways involved, while only a full metagenome sequencing approach coupled with metabolomics could inform us on the functions truly active in the gut microbiota of these genetically divergent broilers.

## Supporting information

S1 Fig NMDS representation of Bray-Curtis distances between samples calculated from OTU counts, according to the sex and represented in each intestinal segment. Ca. caeca; Il. ileum; Je. jejunum.
(TIF)

S2 Fig NMDS representation of Bray-Curtis distances between samples calculated from OTU counts, according to the pen and represented in each intestinal segment. Ca. caeca; Il. ileum; Je. jejunum.
(TIF)

S1 Table Ingredient composition and nutrient composition of the diet. (DOCX)

S2 Table List of KO differentially abundant between AMEn+ and AMEn- samples (excel file). (XLSX)

S3 Table Animals distribution among each pen and sex. (XLSX)

S4 Table Effect of sex and pen on microbiota alpha and beta-diversity diversity. (XLSX)

S5 Table Metadata and data used to produce figures. (XLSX)

S1 File NC3RsARRIVE guidelines checklist. (DOCX)
