# Supplementary figures and images for: Broilers divergently selected for digestibility differ for their digestive microbial ecosystems
Source: PLoS One. 2020 May 18;15(5):e0232418. doi: 10.1371/journal.pone.0232418 (PMC7233591; doi:10.1371/journal.pone.0232418)

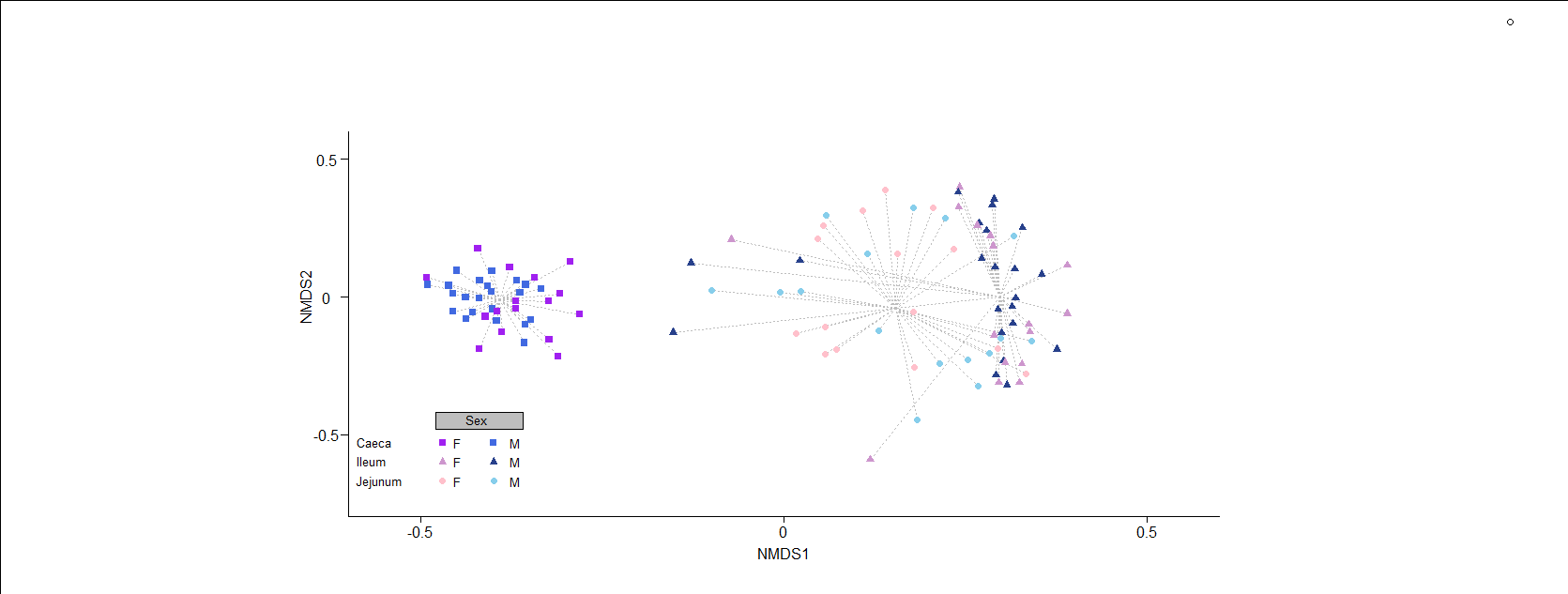

Supplement: S1 Fig — Ca. caeca; Il. ileum; Je. jejunum. (TIF) [file pone.0232418.s001.tif]

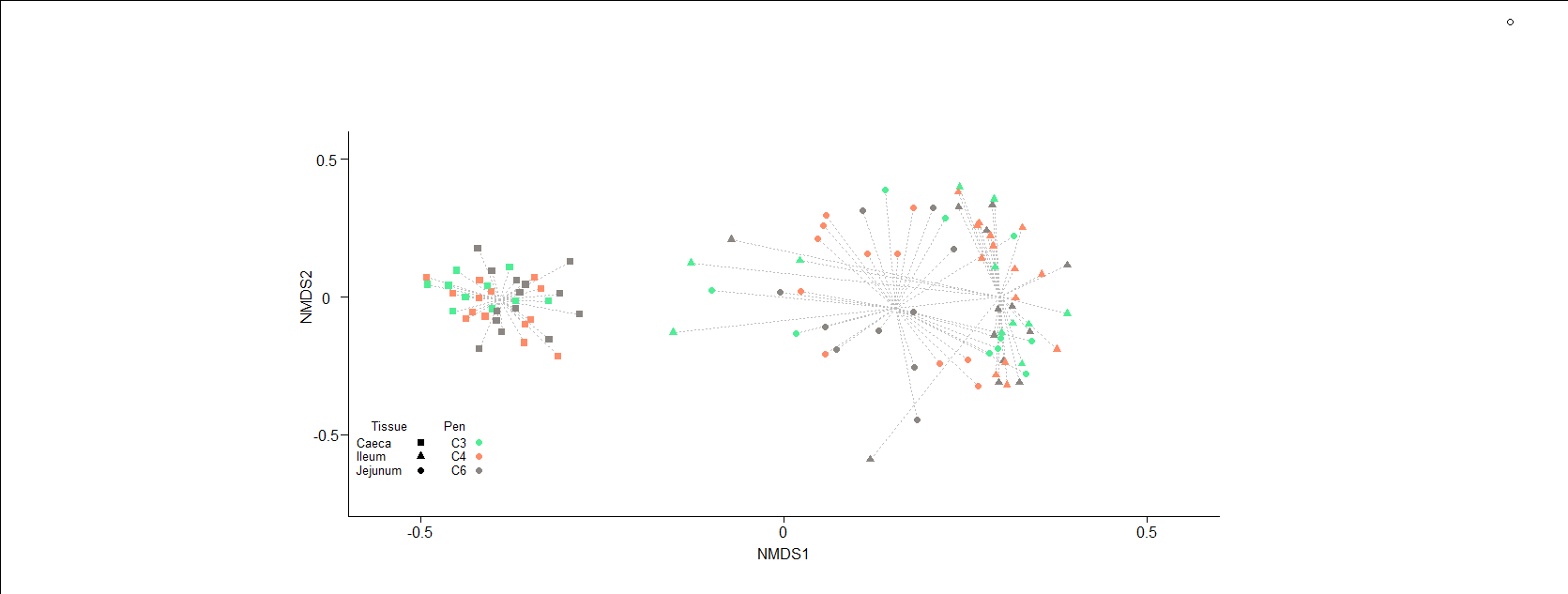

Supplement: S2 Fig — Ca. caeca; Il. ileum; Je. jejunum. (TIF) [file pone.0232418.s002.tif]
