# Supplementary material for: Broilers divergently selected for digestibility differ for their digestive microbial ecosystems
Source: PLoS One. 2020 May 18;15(5):e0232418. doi: 10.1371/journal.pone.0232418 (PMC7233591; doi:10.1371/journal.pone.0232418)
Supplement: S1 Table — (DOCX) [file pone.0232418.s003.docx]

Table S1: composition of the diet given to animals

| **Ingredients** | (%, as fed basis) |
| --- | --- |
| Corn | 6.04 |
| Rialto wheat | 52.50 |
| Soya oil | 6.00 |
| Soybean cake 48 | 28.40 |
| Corn gluten 60 | 3.10 |
| Calcium carbonate | 1.34 |
| Dicalcium phosphate | 1.58 |
| Sodium | 0.30 |
| Oligo-vitamin premix | 0.35 |
| DL Methionine | 0.12 |
| L-Lysine 78 | 0.22 |
| Anticoccidial (Clinacox)^1^ | 0.02 |
| **TOTAL** | **100.00** |

^(1)^ Diclazuril (non-ionophore synthetic product; coccidiostat; without any effect reported on the gut microbiota)
