# Supplementary material for: Broilers divergently selected for digestibility differ for their digestive microbial ecosystems
Source: PLoS One. 2020 May 18;15(5):e0232418. doi: 10.1371/journal.pone.0232418 (PMC7233591; doi:10.1371/journal.pone.0232418)

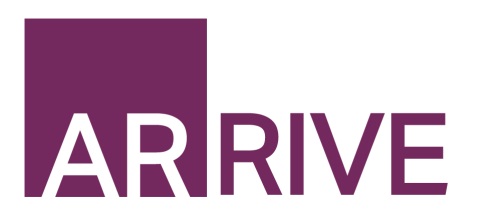


The ARRIVE Guidelines Checklist

Animal Research: Reporting In Vivo Experiments

Carol Kilkenny^1^, William J Browne^2^, Innes C Cuthill^3^, Michael Emerson^4^ and Douglas G Altman^5^

*^1^The National Centre for the Replacement, Refinement and Reduction of Animals in Research, London, UK, ^2^School of Veterinary Science, University of Bristol, Bristol, UK, ^3^School of Biological Sciences, University of Bristol, Bristol, UK, ^4^National Heart and Lung Institute, Imperial College London, UK, ^5^Centre for Statistics in Medicine, University of Oxford, Oxford, UK.*

|  | | ITEM | RECOMMENDATION | Section/ Paragraph |
| --- | --- | --- | --- | --- |
| 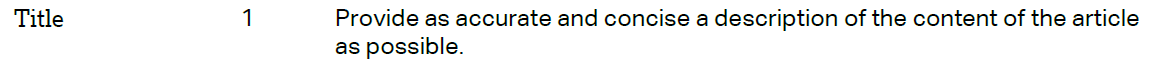 | | | **Title** |  |
| 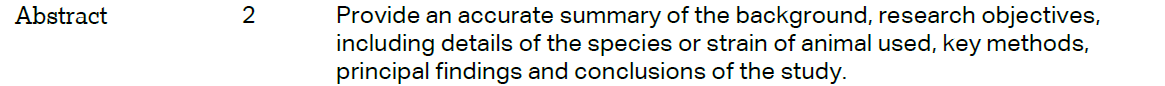 | | | **Abstract**  **Background : ok**  **Objectives : ok**  **Animal species: ok**  **Methods : ok**  **Principal results : ok**  **Conclusion : ok** |  |
| INTRODUCTION | | |  |  |
| 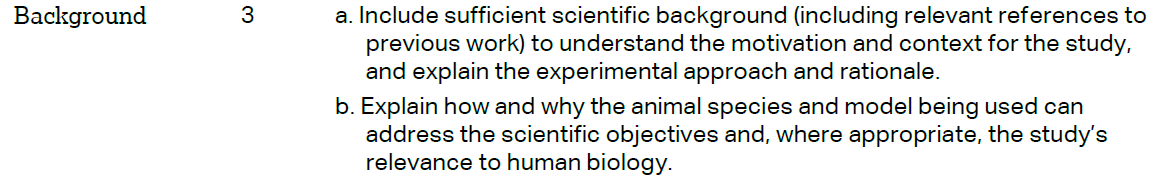 | | | a. **Introduction section: paragraphs 1, 2, 3**  **b. Introduction section: paragraphs 3, 4** |  |
| 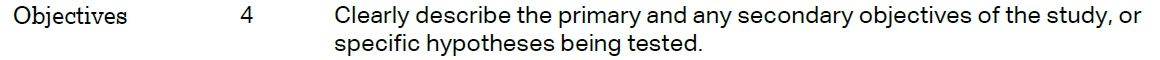 | | | **Introduction, paragraph 4** |  |
| METHODS | | |  |  |
| 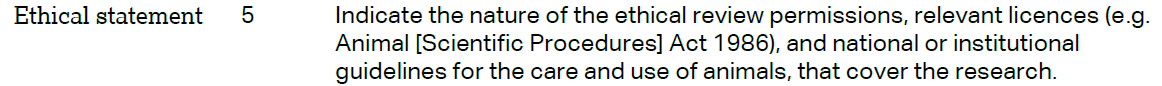 | | | **Materials and Methods/Animal rearing** |  |
| 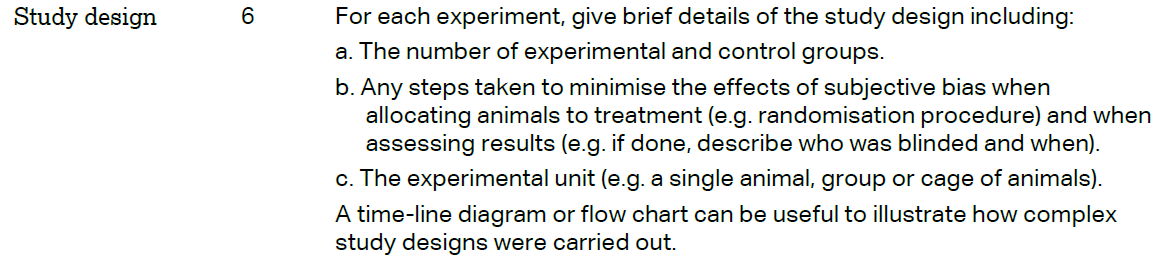 | | | a. **Materials and Methods: Sample collection & Results: Digestibility phenotyping and establishment of contrasted groups**  b. **Materials and Methods: Animal rearing:**  **‘animal were equally distributed into 3 independent pens in the same building’**  **c. experimental unit: single animal**  **Materials and Methods/Sample collection** |  |
| 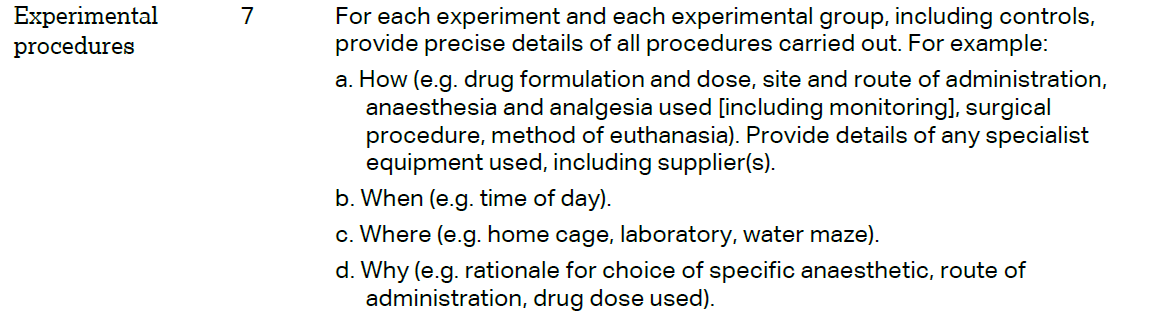 | | | **a. Materials and Methods: Sample collection**  **b. Materials and Methods: Sample collection**  **c-d. Materials and Methods: Animal rearing** |  |
| 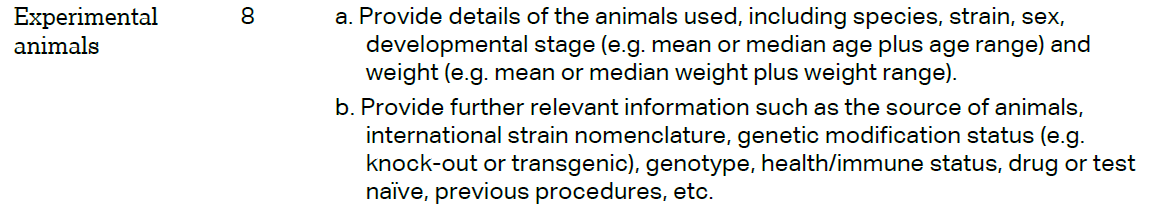 | | | **a. Materials and Methods: Sample collection**  **b. Materials and Methods: Animal rearing and Sample collection** |  |

The ARRIVE guidelines. Originally published in *PLoS Biology*, June 2010^1^

| 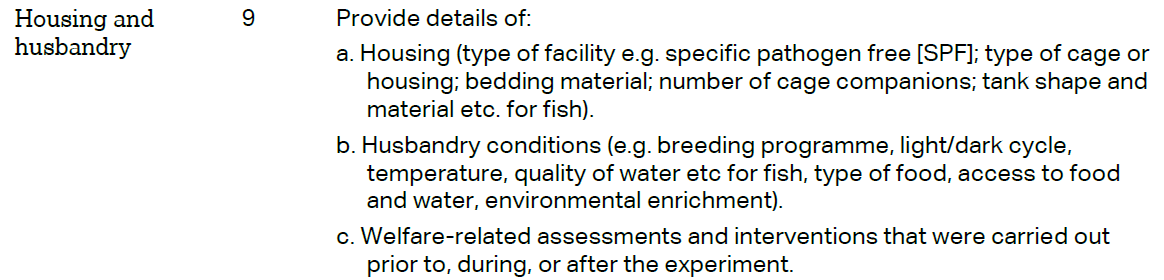 | a.-b.-c. **Materials and Methods: Animal rearing** |  |
| --- | --- | --- |
| 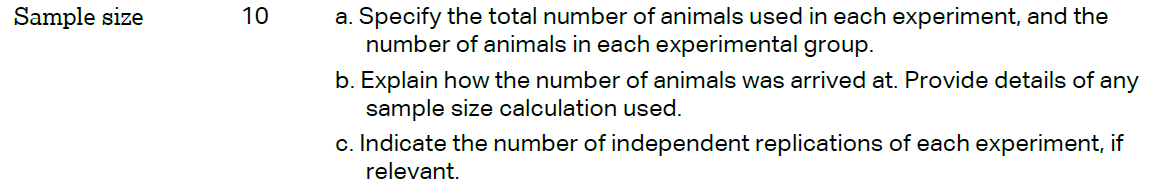 | **a. Materials and Methods: Sample collection & Results: Digestibility phenotyping and establishment of contrasted groups**  **b. We calculated the number of animals (30 per group) according to previous results (Mignon-Grasteau et al, PLOSONE 2015), in order to confirm differences in bacterial species abundances between groups of animals with contrasted levels of digestive efficiency.**  **c. Not relevant.** |  |
| 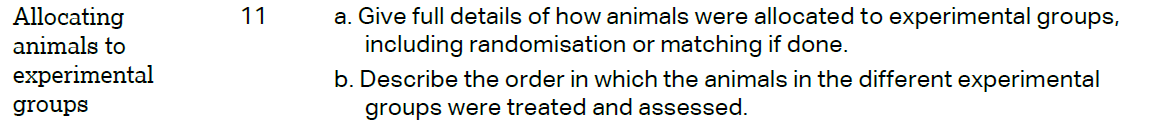 | **Materials and Methods: Animal rearing** |  |
| 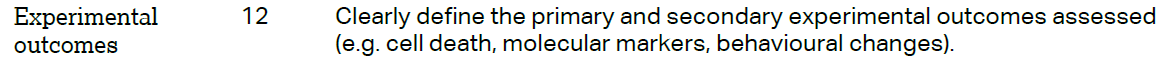 | **Materials and Methods: Sample collection and DNA extraction, amplification and sequencing** |  |
| 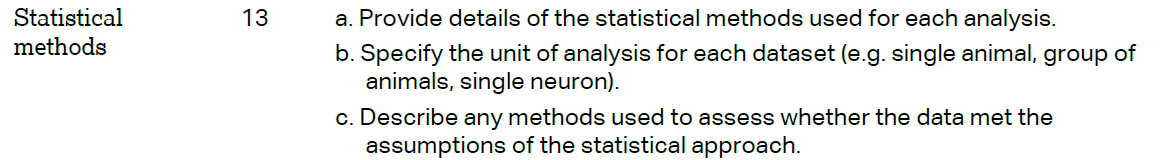 | **Materials and Methods: Statistical analyses: Exploratory analyses and Assessing differentially abundant OTUs between digestibility groups** |  |
| RESULTS |  |  |
| 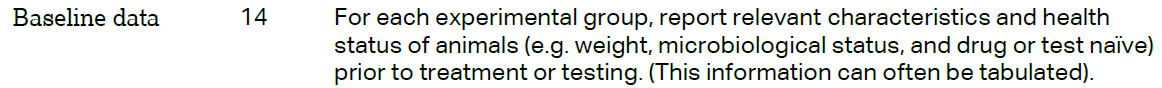 | Results: Digestibility phenotyping and establishment of contrasted groups |  |
| 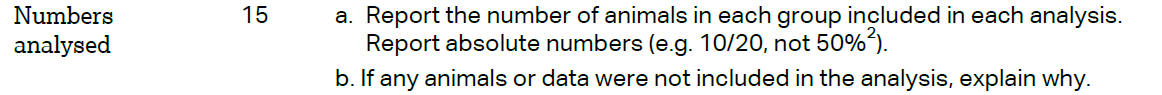 | a. Results: Digestibility phenotyping and establishment of contrasted groups  b. Results: Exploratory microbiota analysis over all intestinal segments |  |
| 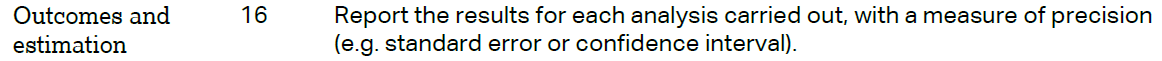 | Results: Microbiota differences among the three intestinal segments |  |
| 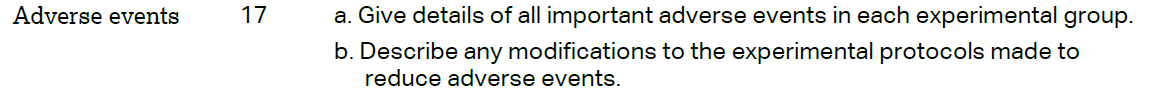 | Not relevant. |  |
| DISCUSSION |  |  |
| 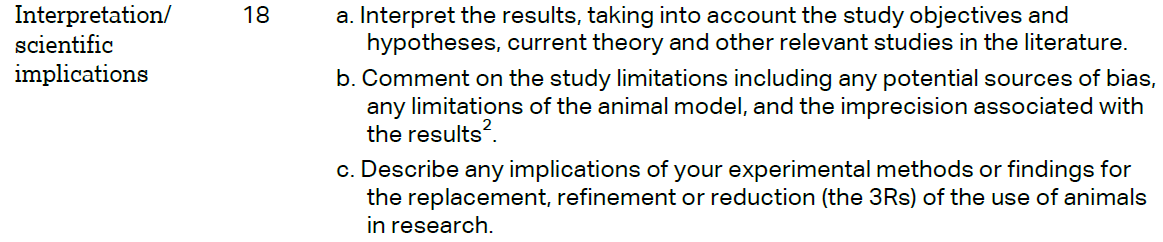 | a. Discussion; Exploratory analysis + Influence of the digestibility level on the gut microbiota + Identification of differentially abundant OTUs + Functional analysis.  b. limitation: Discussion: Functional analysis/ paragraph 2.  c. Not relevant here. |  |
| 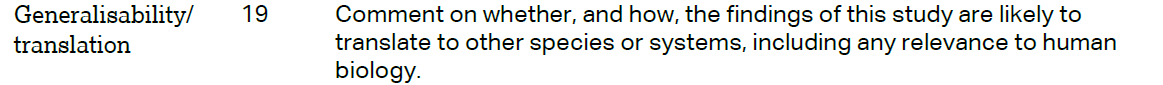 | Not relevant here, since chicken is a model neither for human, nor for any other species. |  |
| 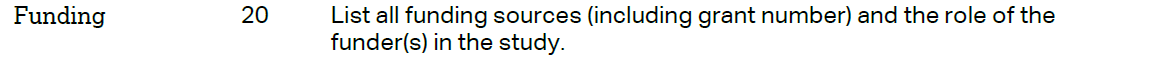 | | Ending section/ Acknowledgements |


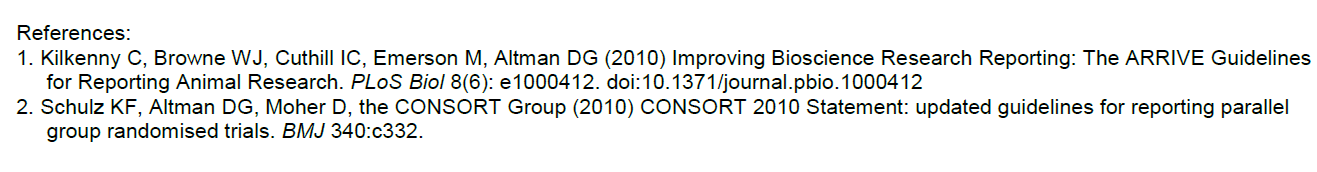

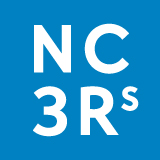

Supplement: S1 File — (DOCX) [file pone.0232418.s008.docx]
